# Metachronous triple cancer associated with Peutz–Jeghers syndrome treated with curative surgery: a case report

**DOI:** 10.1186/s40792-018-0492-6

**Published:** 2018-08-01

**Authors:** Toru Yoshikawa, Tomoyuki Abe, Hironobu Amano, Keiji Hanada, Tomoyuki Minami, Tsuyoshi Kobayashi, Shuji Yonehara, Masahiro Nakahara, Hideki Ohdan, Toshio Noriyuki

**Affiliations:** 1 Department of Surgery, Onomichi General Hospital, 1-10-23, Onomichi, Hiroshima, 722-8508 Japan; 2 Department of Gastroenterology, Onomichi General Hospital, Onomichi, Hiroshima, Japan; 3 Department of Pathology, Onomichi General Hospital, Onomichi, Hiroshima, Japan; 4 Department of Gastroenterological and Transplant Surgery, Graduate School of Biomedical and Health Sciences, Hiroshima University, Hiroshima, Japan

**Keywords:** Curative surgery, Metachronous cancer, Peutz–Jeghers syndrome

## Abstract

**Background:**

Peutz–Jeghers syndrome (PJS) is an autosomal dominant disorder characterized by mucocutaneous pigmentation and hamartomatous gastrointestinal polyposis. It is well known that individuals with PJS are at an increased risk of cancer in a variety of organs.

**Case presentation:**

Here, we present a patient with PJS who achieved long-term survival by undergoing repeat curative surgery for metachronous triple cancer. Her medical history included hilar cholangiocarcinoma and cervical carcinoma; curative surgery was performed for both conditions. On annual follow-up, the level of carcinoembryonic antigen was elevated at 6.9 ng/ml. Enhanced computed tomography revealed a cystic tumor consisting of mural nodules at the pancreatic head; the maximal diameter was 15 mm. Magnetic resonance imaging clearly demonstrated the tumor with low intensity on T1-weighted images and high intensity on T2-weighted images. Endoscopic ultrasound sonography showed a high echoic tumor at the pancreatic head, which was confirmed as adenocarcinoma by fine-needle aspiration biopsy. The preoperative diagnosis was intraductal papillary mucinous carcinoma (IPMC; T1N0M0, stage IA). Subtotal stomach-preserving pancreaticoduodenectomy was performed and the final diagnosis was IPMC, stage 0 (TisN0M0).

**Conclusions:**

Aggressive surgery for metachronous triple cancer resulted in good long-term prognosis. Continuous and systematic follow-up would allow the detection of malignancy at an early stage and make treatment with curative surgery possible.

## Background

Peutz–Jeghers syndrome (PJS) is an autosomal dominant disorder with variable inheritance. Characteristics of this disorder are hamartomatous polyps in the gastrointestinal tract and pigmented mucocutaneous lesions [1]. This disorder is also characterized by an increased risk of gastrointestinal and non-gastrointestinal cancer. Accumulative risk for the 15 to 64 years age group was estimated to be approximately 90% for cancer of any organ [1, 2]. Gastrointestinal cancer is known to occur in 40% of PJS cases, and non-gastrointestinal cancer in 10–50% of cases [2]. The etiology of PJS, however, remains unclear. Previous research has suggested that mutation of the *STK11* gene could be responsible for PJS [2]. *STK11* is a tumor suppressor gene that encodes a serine/threonine kinase. Studies have shown that the loss of STK11 protein kinase activity is associated with the occurrence and development of tumors [2–5].

Herein, we present a case in which metachronous triple cancer was curatively resected, leading to a good prognosis. We also provide a review of the relevant scientific and clinical literature that have been published in the English language.

## Case presentation

**Fig. 1:**
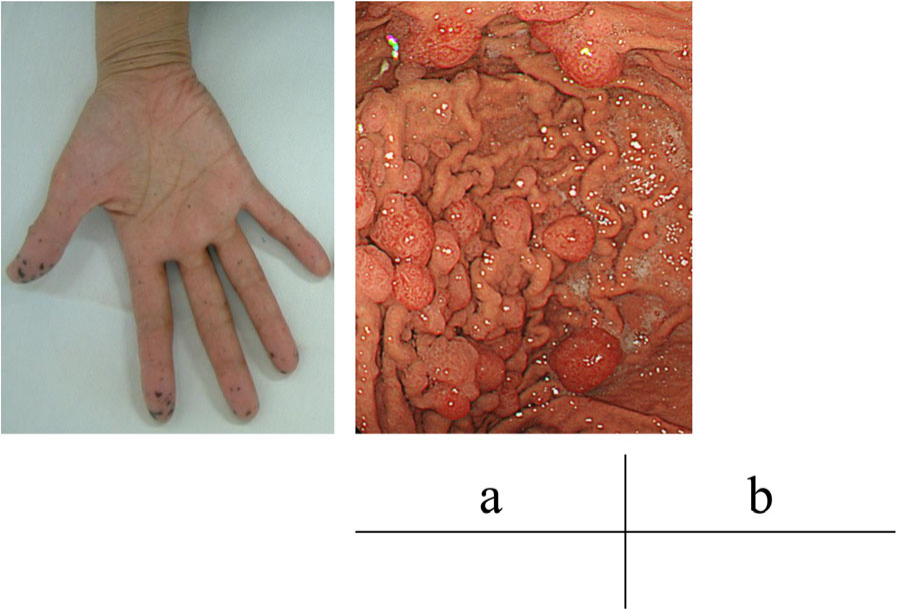
Pigmentation in the hand (**a**) and hamartomatous polyps of the stomach (**b**)

**Fig. 2:**
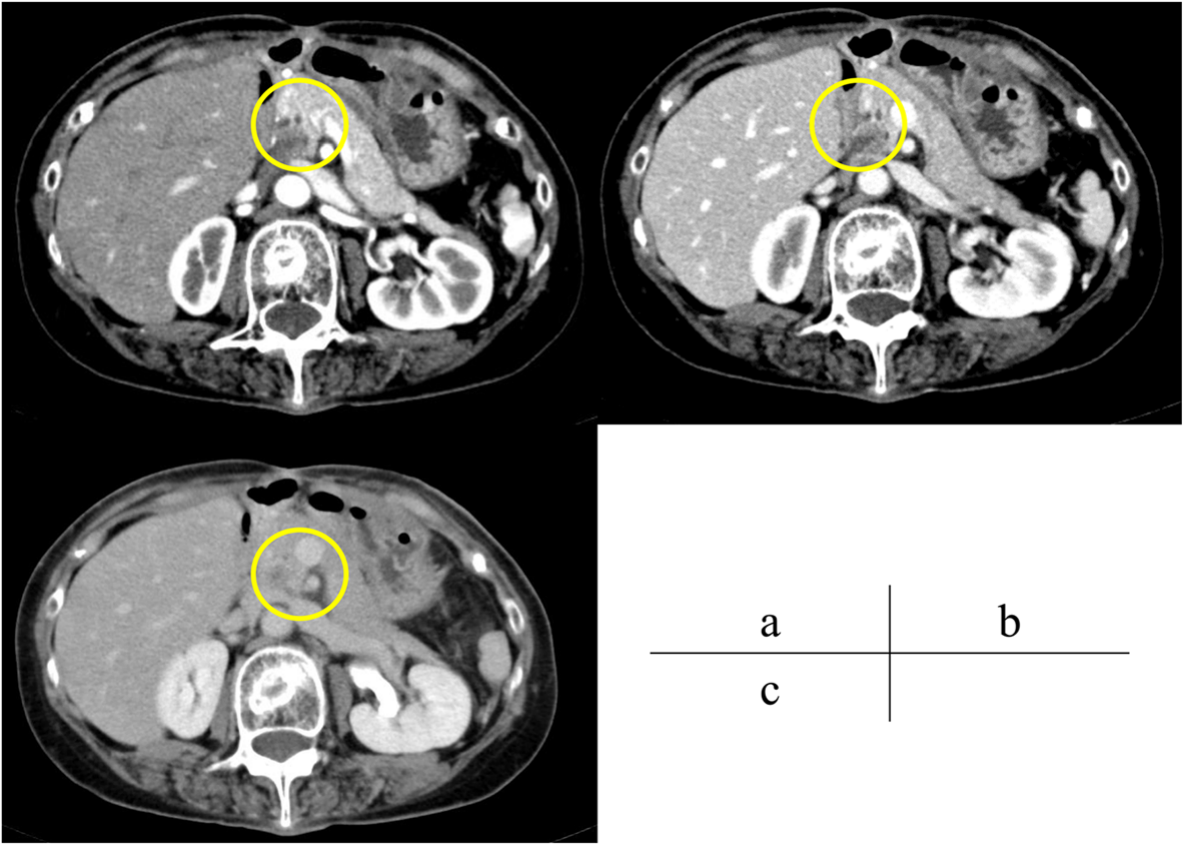
Dynamic abdominal computed tomography (CT) demonstrating a slightly enhanced round tumor at the pancreatic head (**a**, yellow circle). Tumor enhancement was evident from the portal phase to the equal phase (**b**, **c**)

**Fig. 3:**
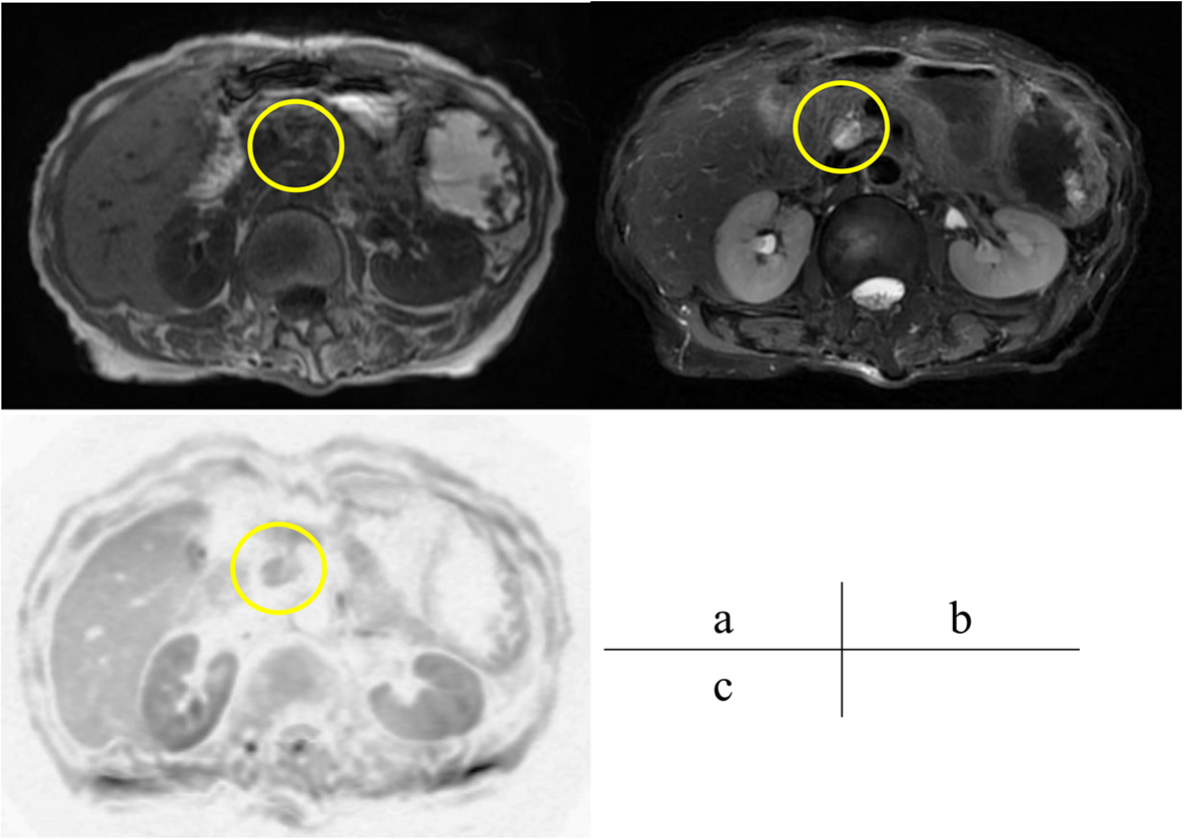
Magnetic resonance imaging (MRI) showing the patient’s tumor with low-intensity on T1-weighted images (**a**, yellow circle), heterogeneous high intensity (**b**) on T-2 weighted images, and high intensity on diffusion-weighted images (**c**)

**Fig. 4:**
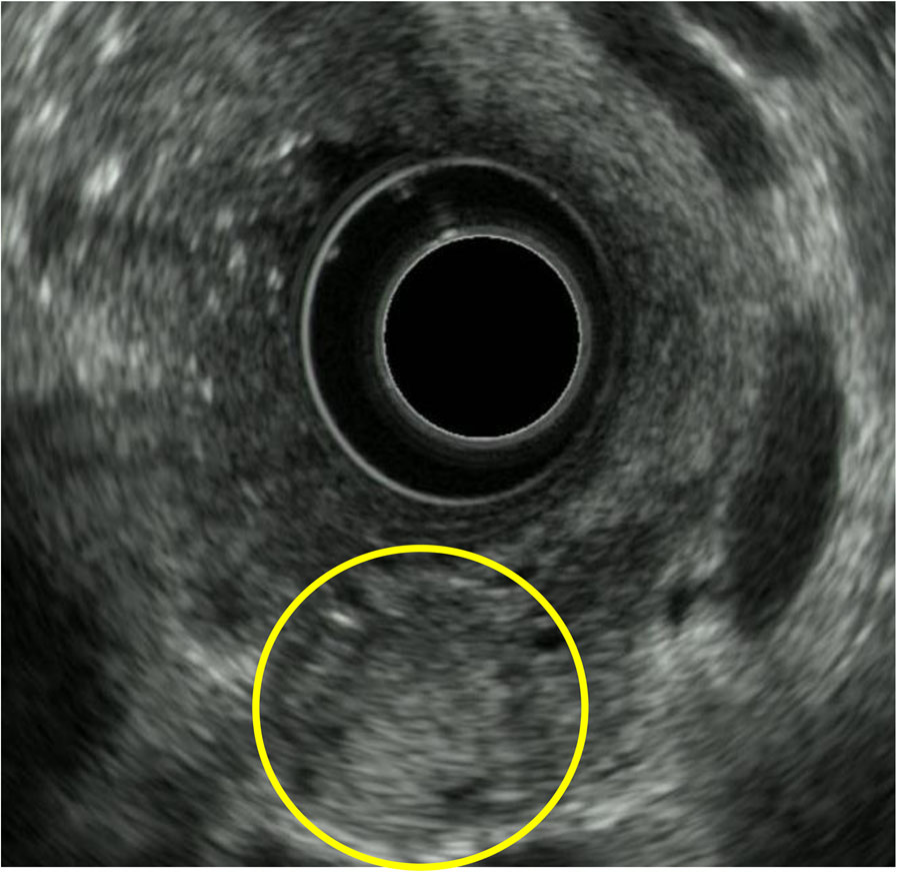
Endoscopic ultrasound sonography showing the high echoic tumor at the pancreatic head

**Fig. 5:**
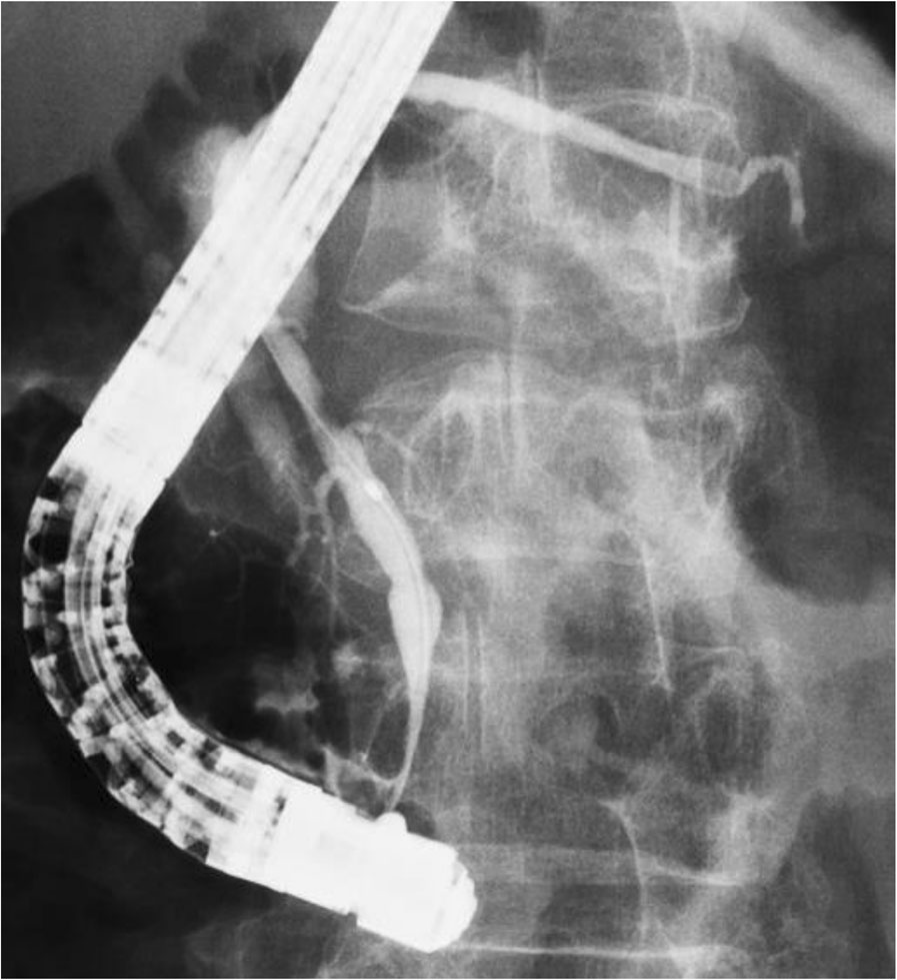
Endoscopic retrograde cholangiopancreatography showed no dilation of the main pancreatic duct and its branch. No mucinous production was detected during any of the procedures

A 62-year-old woman was admitted to Onomichi General Hospital in 2017 with a suspected pancreatic tumor. Her medical history included hilar cholangiocarcinoma (poorly differentiated adenocarcinoma, intermediate type, INFß, pat Bp, ly1, v0, pn0, hinf2, hm0, dm0, em2, 3 cm × 2 cm × 2 cm, T3N0M0 stage IIA, UICC version 6) at the age of 53 years and cervical cancer at the age of 55 years. Each of these was curatively resected. PJS was diagnosed by the presence of hamartomatous polyps in the gastrointestinal tract and melanin pigmentation on the hands 20 years ago (Fig. 1a, b). Family history revealed that her son was also diagnosed with PJS. There was no chief complaint. Level of the tumor marker carcinoembryonic antigen (CEA) was elevated at 6.7 ng/ml; squamous cell carcinoma antigen, carbohydrate antigen 19–9, and laboratory data were within the normal limits. Contrast-enhanced computed tomography (CT) revealed a cystic tumor consisting of mural nodules at the pancreatic head; the maximal diameter was 15 mm. The tumor border was enhanced in the early phase, and the inner portion of the tumor showed low density (Fig. 2a). Tumor enhancement was prolonged in the delayed phase (Fig. 2b, c). Magnetic resonance imaging (MRI) showed the tumor with low intensity on T1-weighted images, high intensity on T2-weighted images, and heterogeneously high intensity on diffusion-weighted images (Fig. 3a–c). Endoscopic ultrasound sonography (EUS) showed a high echoic tumor at the pancreatic head (Fig. 4). Fine-needle aspiration biopsy confirmed adenocarcinoma. Endoscopic retrograde cholangiopancreatography showed no dilation of the papilla of Vater, or mucin production. There was no connection between the tumor and the main pancreatic duct (Fig. 5). The preoperative diagnosis was intraductal papillary mucinous carcinoma (IPMC; T1N0M0, stage IA). Subtotal stomach-preserving pancreaticoduodenectomy was duly performed (operation time, 697 min; bleeding volume, 1200 ml). The pathological diagnosis was IPMC, and the final tumor stage was TisN0M0, stage 0. The patient was subsequently discharged without any complications 20 days after the surgery. There was no recurrence over the 11 month follow-up period.

## Discussion

Giardiello et al. were the first to demonstrate that patients with PJS have an extremely high risk of cancer (approximately 90%) during their lifetime [1, 2]. There are several reports of cases involving double cancer with PJS; however, to the best of our knowledge, metachronous triple primary cancer associated with PJS treated with curative surgery has not yet been reported [6, 7]. These patients are at increased risk from a range of gastrointestinal cancers, including the esophagus, stomach, small intestine, colon, and pancreas [1, 8, 9]. Non-gastrointestinal cancers have also been reported to occur in a high proportion of PJS patients, including cancer of the breast, ovary, cervix, and sex cord [1, 9].

Surveillance programs for malignant tumors have been proposed by several clinical guidelines or recommendations [1, 3, 10]. However, surveillance strategies can vary according to organ-specific cancer occurrence [1]. The approximate lifetime risk of uterine and cervical cancer in PJS patients is 9 and 10%, respectively. Screening by pelvic examination using the Papanicolaou test every year, beginning at the age of 21 years, is strongly recommended, not only in ordinal females but also in PJS patients. In addition, adenoma malignum, which has a dismal prognosis, is more likely to occur in patients with PJS than in healthy females. In addition, transvaginal ultrasound, with the carbohydrate antigen 125 test, is recommended for ovarian cancer screening, beginning at the age of 25 years [1, 11]. Pancreatic cancer is one of the most common tumors affecting PJS patients, with a lifetime risk of approximately 30% [4, 10, 12]. A recent consensus conference of the International Hereditary Pancreatitis Study Group recommended screening for pancreatic neoplasms, especially in patients with PJS. EUS, CT, and MRI are the most commonly used techniques for pancreatic cancer screening. Recent reports have stated that EUS is the most reliable technique for detecting early pancreatic cancer [13]. Pancreatic cancer surveillance, on an annual or biennial basis, between the ages of 25 and 30 years is recommended by several reports [14, 15]. Although the lifetime risk for carcinoma of the biliary tract has not yet been clarified, some important reports have documented these carcinomas and estimated their frequency to be about one-quarter of that of pancreatic carcinoma [1]. Surveillance of the biliary tract has rarely been documented, but radiographic surveillance, performed synchronously with tests for the pancreas, seems to be a good practical option [9].

Current evidence for appropriate surveillance guidelines is not sufficient, largely because of the rarity of this disease and the lack of accumulated data addressing the efficacy and outcomes of surveillance for patients with PJS. In our particular case, tumor markers were checked every 3 months, and CT was performed every 6 months, as a follow-up schedule following cholangiocellular carcinoma resection. Annual gastrointestinal endoscopy and the Papanicolaou test were performed every 6 months as a follow-up after cervical cancer resection. However, the optimal form of surveillance still remains unclear. The CAPS 3 study and other reports have advocated that patients with PJS should begin surveillance at least by 40 years of age and that patients without PJS, but with high risk for pancreatic ductal adenocarcinoma (PDAC) should begin surveillance by at least 50 years of age [16, 17]. PDAC is one of the most lethal malignancies; as such, special attention should be paid to the detection of PDAC at an early stage. In order to detect cases of early stage PDAC, Hanada et al. recommended that EUS and Magnetic resonance cholangiopancreatography should play an important role, rather than dynamic CT [13].

Multiple primary malignancy (MPM) is defined as two or more malignancies, without any relationship between the tumors, occurring in the same individual either simultaneously or metachronously [18]. Several factors could lead to a deterioration in host immune function, such as chemotherapy or radiotherapy after the first primary malignancy. However, not only host immune deficiency can lead to cancer; it is possible that environmental factors leading to carcinogen exposure could also lead to cancer. This may lead to the development of MPM at some point during the lifetime [19–22]. Multiple metachronous malignancies are frequently detected in hematological, lung, thyroid, breast, skin, and genitourinary malignancies [23, 24]. Liu et al. investigated the etiological factors, clinical characteristics, diagnosis, treatment strategies, and prognosis of MPM and demonstrated that curative surgery has a strong impact on long-term prognosis [22].

Our current patient had hilar cholangiocarcinoma and pancreatic cancer; these are conditions that are known to have a poor prognosis, although patients have survived for more than 9 years after surgery for hilar cholangiocarcinoma. Regardless of the dismal prognosis after curative surgery for biliary and pancreatic cancer, good long-term prognosis was achieved in our case. Repeat curative surgery could therefore have a strong impact on the long-term prognosis of patients with PJS. There are no standard guidelines for the management of PJS and MPM at present. However, we recommend that physicians take into consideration the type of malignancies, progression of disease, response to therapy, and the general condition of patients.

## Conclusions

Herein, we present a case of repeat curative surgery for metachronous triple cancer associated with PJS. Aggressive surgery should be considered, even in patients with a high risk of malignancy; such action should lead to good long-term prognosis.

## Abbreviations

CEA: Carcinoembryonic antigen
CT: Computed tomography
EUS: Endoscopic ultrasound sonography
IPMC: Intraductal papillary mucinous carcinoma
MPM: Multiple primary malignancy
MRI: Magnetic resonance imaging
PDAC: Pancreatic ductal adenocarcinoma
PJS: Peutz–Jeghers syndrome
